# Nexilin Regulates Oligodendrocyte Progenitor Cell Migration and Remyelination and Is Negatively Regulated by Protease-Activated Receptor 1/Ras-Proximate-1 Signaling Following Subarachnoid Hemorrhage

**DOI:** 10.3389/fneur.2018.00282

**Published:** 2018-04-25

**Authors:** Qiang Li, Hengli Zhao, Pengyu Pan, Xufang Ru, Shilun Zuo, Jie Qu, Bin Liao, Yujie Chen, Huaizhen Ruan, Hua Feng

**Affiliations:** ^1^Department of Neurosurgery, Southwest Hospital, Third Military Medical University, Chongqing, China; ^2^Department of Neurobiology, College of Basic Medical Sciences, Third Military Medical University, Chongqing, China

**Keywords:** subarachnoid hemorrhage, nexilin, oligodendrocyte progenitor cell, Ras-proximate-1, remyelination

## Abstract

Progressive white matter (WM) impairments caused by subarachnoid hemorrhage (SAH) contribute to cognitive deficits and poor clinical prognoses; however, their pathogenetic mechanisms are poorly understood. We investigated the role of nexilin and oligodendrocyte progenitor cell (OPC)-mediated repair in a mouse model of experimental SAH generated *via* left endovascular perforation. Nexilin expression was enhanced by the elevated migration of OPCs after SAH. Knocking down nexilin by siRNA reduced OPC migration both *in vitro* and *in vivo* and abridged WM repair. In contrast, the protease-activated receptor 1 (PAR1), Ras-proximate-1 (RAP1) and phosphorylated RAP1 (pRAP1) levels in WM were elevated after SAH. The genetic inhibition of PAR1 reduced RAP1 and pRAP1 expression, further enhancing nexilin expression. When delivered at an early stage at a concentration of 25 µg/kg, thrombin receptor antagonist peptide along with PAR1 knockdown rescued the down-regulation of myelin basic protein and improved remyelination at the later stage of SAH. Our results suggest that nexilin is required for OPC migration and remyelination following SAH, as it negatively regulates PAR1/RAP1 signaling, thus providing a promising therapeutic target in WM repair and functional recovery.

## Introduction

White matter (WM) occupies over 50% of the human brain and is composed mainly of axons and myelin, both thought to be vulnerable to hemorrhagic stroke, including subarachnoid hemorrhage (SAH) (1). Imageology evidence in SAH patients and animal models revealed that WM lesions in the acute phase are associated with poor outcomes (2, 3). In the clinic, SAH patients who initially present with low World Federation of Neurosurgical Societies (WFNS) grades and hydrocephalus (which may be the reason for WM damage) can be efficiently treated with extraventricular drainage and often show rapid improvement, but patients with high-WFNS grades and low-Glasgow Coma Scale scores rarely have good recoveries or even only moderate disabilities (4). Moreover, autopsied cases of SAH showed remarkable congestion and edema in the deep part of frontal WM and severe diffuse axonal injury (5). Previous autopsy studies also found apparent WM lesions separate from prevalent cortical and hypothalamic lesions (6). However, investigations of WM injuries are not sufficient in SAH. Currently, there are no effective treatments for the demyelination-induced progressive deterioration of neurological function after SAH. Fortunately, a re-ensheathment mechanism has been found to boost functional recovery, as remyelination occurs as stem cells proliferate, migrate, and differentiate into mature functional cells (7). This mechanism involves amplification of the oligodendrocyte progenitor cell (OPC) pool and cell recruitment to the lesion area to benefit remyelination and functional recovery regardless of whether the amplification occurs by increasing OPC proliferation (8–10).

After brain injury, OPC migration is an extremely complex, precise, and widespread event that occurs across long distances to colonize the entire brain (11). Different mechanisms have been shown to direct the migration of OPCs, including extracellular chemotropic cues (laminin, N-cadherin, merosin, semaphorin 7F, tenascin-C, etc.) and secreted adhesion molecules [platelet-derived growth factor (PDGF), vascular endothelial growth factor, semaphorin 3A (SEMA3A), chemokine (C-X-C motif) ligand 1 (CXCL1), etc.] (12, 13). However, how microtubule rearrangement interacts with F-actin to affect the extension of cell protrusions necessary for crawling is poorly understood, but this interaction is necessary for OPC migration (14, 15). Nexilin, a newly identified F-actin binding protein, was reported to stimulate cell migration and adhesion (16, 17). Furthermore, nexilin is enriched in both cardiomyocytes and brain neural and glial cells (18). Therefore, we speculated that nexilin can regulate OPC migration by binding F-actin when expressed in OPCs.

A previous study showed that the transcription factor protease-activated receptor 1 (PAR1) is a key molecule in F-actin-dependent migration, and thrombin receptor PAR1 can activate Ras-proximate-1 (RAP1) (19). The thrombin-activated PAR1/RAP1 signaling pathway is important for platelet aggregation, as it partially increases cell adhesion while reducing migration and thus plays roles in hemostasis and thrombosis (20). Another study showed that PAR1 activated the Sav1-bound protein kinases Lats1/Lats2 and phosphorylated RAP1 (pRAP1), thereby negatively controlling T cell adhesion and migration, a program crucial for immune surveillance and response (21). These results suggest that the PAR1/RAP1 signaling pathway is a potential mechanism that regulates cell migration. In fact, PAR1 is expressed in OPCs and has been proven to suppress their differentiation (22). The chemical inhibition or genetic deletion of PAR1 resulted in earlier spinal cord myelination onset, inducing substantially more Olig2-positive oligodendrocytes, more myelinated axons and higher proteolipid protein levels (23). In addition, a thrombin inhibitor has been shown to prevent SAH vasospasms, suggesting that PAR1 signaling could be a promising therapeutic target (24). These data indicated that the PAR1/RAP1 signaling pathway may be a critical switch controlling myelination in subarachnoid hemorrhagic WM injuries.

Based on these considerations, we hypothesized that nexilin is an indispensable cue promoting OPC proliferation, migration, and remyelination in mice with SAH and that this process might be mediated by PAR1–RAP1 signaling. Given the translational consideration, we also studied whether a thrombin receptor antagonist peptide (TRAP) promotes WM repair by acting on nexilin.

## Materials and Methods

### Animals

We obtained 103 healthy adult (3-month-old) male C57BL/6 mice weighing 25–30 g and 15 newborn (postnatal days 1–3) C57BL/6 mice from the Third Military Medical University animal center (Chongqing, China). The mice were housed at two mice per cage (Macrolon-II-cages, 18 cm × 24 cm × 14 cm) in an environment with a 12-h light/dark cycle and allowed free access to food and water throughout the experimental period. All mice were randomly assigned to the different experimental groups. All the experimental procedures were approved by the Ethics Committee of Southwest Hospital, performed in accordance with guidelines established by the National Institutes of Health Guide for the Care and Use of Laboratory Animals, and reported following ARRIVE (Animal Research: Reporting of *In Vivo* Experiments) guidelines.

### Induction of the SAH Model

The mouse endovascular perforation model of SAH was induced as reported previously (25). Briefly, mice were anesthetized with 5% chloral hydrate (350 mg/kg, intraperitoneal). A sharpened 5-0 monofilament nylon suture was inserted rostrally into the left internal carotid artery from the external carotid artery stump and perforated the bifurcation of the anterior and middle cerebral arteries. Sham-operated mice underwent the same procedure except the suture was withdrawn without puncture. The surgery wound was closed with suture clips, and Xylocaine was delivered subcutaneously near the wound to reduce post-operative pain. Physiological parameters of experimental mice were monitored during surgery by a Small Animal Vital Signs Monitor (MouseOx, Wallkill, NY, USA). Blood pressure and heart rate were non-invasively monitored during operation from the tail. All animals regained consciousness within 30 min, and only mice with an SAH demonstrated an ipsilateral turning bias. Their ability to eat, drink, and groom themselves was transitory impaired, and 13 mice died because of surgery or SAH induction (excessive bleeding).

The severity of SAH was blindly assessed in all animals after they were sacrificed as previously described (26). Briefly, the subarachnoid cistern was divided into six segments, and each segment was allotted a grade from 0 to 3 as follows; grade 0 = no subarachnoid blood; grade 1 = minimal subarachnoid blood; grade 2 = moderate blood clot with recognizable arteries; and grade 3 = blood clot obliterating all arteries within the segment. Each animal received a total score by summing all the scores, and only SAH mice with moderate hemorrhage (scores 8–12) were included in the experiments.

### siRNA and Drug Administration *In Vivo*

According to methods described previously (27), an intracerebroventricular injection was performed. Briefly, the mice were placed on a stereotaxic apparatus (Rwdmall, Guangzhou, China) under anesthesia with 5% chloral hydrate (350 mg/kg, intraperitoneal), and the bregma point was then exposed. A burr hole was drilled into the bone of the left hemisphere at the following coordinates: 1 mm lateral, 0.2 mm posterior, and 2.2 mm below the horizontal plane of the bregma. Next, a 2 µL volume of specific siRNAs and drugs was delivered to the lateral ventricle with a Hamilton syringe (Hamilton Company, Reno, NV, USA). These siRNAs and drugs included 1 µg/µl nexilin siRNA, PAR1 siRNA or the respective scramble siRNA (Santa Cruz, CA, USA), 62.5 µg/ml (5 µg/kg), or 312.5 µg/ml (25 µg/kg) TRAP (Bachem, Bubendorf, Switzerland) and an equal volume of saline as the vehicle. The injection was performed twice at 12 and 24 h before SAH to enhance the silencing effect.

### Neurological Scoring

As previously reported, neurological functions were evaluated by a modified neurological severity scale (mNSS) method at 1, 3, 5, 7, 9, 12, and 14 days after SAH (28). The mean neurological score was graded by two blinded observers.

### Electron Microscopy

As reported by Chia-Yu Yeh et al., the mice were transcardially perfused with a fixation buffer containing 2.5% glutaraldehyde and 4% paraformaldehyde (PFA) (29). Samples of the white mater middle segment ipsilateral to the occluded carotid were obtained and stored in the same buffer for 48 h at 4°C. The samples were then post-fixed in 1% osmium tetroxide, dehydrated in cold ethanol, infiltrated, and embedded in Epon812 resin. Sections (120 nm thick) were then collected, placed on grids, and stained with uranyl acetate and lead citrate. The grids were then imaged using a transmission electron microscope. Myelinated axon quantification was performed on 12 random images per animal from different scan fields. G-ratios (ratio of the axon diameter to the axon + myelin sheath diameter) were calculated using ImageJ software for at least 60 fibers per animal.

### Cell Culture

Primary OPC cultures were obtained from C57 mice on postnatal day 1 as described previously (30). First, cerebral hemispheres from 1-day-old mice were mechanically dissociated and plated on poly-d-lysine-coated flasks in DMEM/F12 (1:1 v/v; Gibco, Grand Island, NY, USA) supplemented with 10% FBS (Invitrogen, USA). After 4 h, the medium was changed, and the cells were grown in DMEM/F12 supplemented with insulin (5 µg/ml), apo-transferrin (50 µg/ml), sodium selenite (30 nM), D-biotin (10 mM), and 10% FBS (Gibco). Two-thirds of the culture medium was changed every 3 days. After 14 days, OPCs were purified from the mixed glial culture by the differential shaking and adhesion procedure established by Suzumura and Silberberg (31) and were allowed to grow on poly-d-lysine-coated coverslips in DMEM/F12 supplemented with insulin (5 µg/ml), apo-transferrin (50 µg/ml), sodium selenite (30 nM), 0.1% BSA, progesterone (0.06 ng/ml), and putrescine (16 µg/ml; Sigma-Aldrich). T3 (15 nM) was added to inhibit the differentiation of OPCs. Scramble or nexilin siRNA (2 ng/ml, Santa Cruz) was added and cultured with the OPCs for 1 day prior to analysis.

### Transwell Chamber Assays

As previously reported, primary OPCs were seeded in an 8 µm pore-sized transwell-based Boyden chamber system (Corning Incorporated, NY, USA) to perform the OPC migration assay (32). Scramble or nexilin siRNA (2 ng/ml, Santa Cruz) was added and cultured with the OPCs for 1 day prior to analysis. OPCs were seeded in the upper chamber (pre-coated with poly-d-lysine on both sides) at 100,000 per well and maintained in 200 µl of neurobasal medium supplemented with Ara-C (5 µM, Sangon Biotech, China) to prevent proliferation. PDGF (10 ng/ml, Sangon Biotech) was added to the lower chamber to induce the migration of OPCs for 24 h. The migrated cells on the lower chamber were counted in six randomly selected microscopic fields from each well. Then, these cells were fixed with 4% PFA and subjected to immunofluorescence assessment.

### Immunochemical Staining

For cell staining, primary OPCs were pre-seeded on a confocal 35 mm culture dish (Corning, Shanghai, China). For slice staining, 30 µm coronal brain frozen sections were prepared and subjected to immunochemical staining according to a previously reported protocol (27). Briefly, the cells and sections were first treated with 0.5% Triton X-100 and 3% BSA for 1 h and then incubated with suitable antibodies for 24 h at 4°C. The antibodies included rabbit anti-nexilin (1:100; Abcam, MA, USA), mouse anti-F-actin (1:200; Abcam), mouse anti-NG2 (1:200; Millipore, MA, USA), rabbit anti-NG2 (1:100; Abcam), mouse anti-Olig2 (1:200; Millipore), rabbit anti-pRAP1 (1:200; Millipore), rabbit anti-RAP1 (1:200; Millipore), and rabbit anti-myelin basic protein (MBP) (1:200; Boster, Wuhan, China). The slices were then washed three times in 0.01 M PBS overnight at 4°C and incubated with the appropriate secondary antibody (FITC- or CY3-conjugated goat anti-mouse/rabbit IgG antibody; 1:100; Beyotime Institute of Biotechnology, Haimen, China) for 2 h at room temperature. DAPI (4’,6-diamidino-2-phenylindole dihydrochloride) was counterstained to label nuclei. The unspecific bindings were precluded by negative control: PBS or unrelated primary antibody (e.g., mouse anti-foxf2 antibody in this study) instead of primary antibodies. The stained dishes were observed by a laser confocal imaging system (Z780, Carl Zeiss, Germany). At least four confocal images from different view field with magnification of 200 times were involved in counting NG^2+^Olig^2+^ cells.

### Western Blot Assay

Cultured cells and tissues obtained from the corpus callosum (CC), WM and subventricular zone (SVZ) of the mouse brain after surgery were carefully prepared. To avoid the dephosphorylation and degradation of proteins, tissues were transferred into an ice-cold RIPA buffer solution (pH 7.4, Beyotime) containing a cocktail (Roche, Basel, Switzerland) of protein phosphatase and proteinase inhibitors (50 mM Tris–HCl, 100 mM NaCl, 15 mM sodium pyrophosphate, 50 mM sodium fluoride, 1 mM sodium orthovanadate, 5 mM EGTA, 5 mM EDTA, 1 mM phenylmethylsulfonyl fluoride, 1 µM microcystin-LR, 1 µM okadaic acid, 0.5% Triton X-100, 2 mM benzamidine, 60 µg/ml aprotinin, and 60 µg/ml leupeptin) and ground with a pellet pestle (Kontes glassware, Vineland, NJ, USA). The homogenate was then centrifuged at 15,000 × *g* for 10 min at 4°C, and the supernatant was assayed for total protein concentration using the Bradford Protein Assay Kit (Beyotime). Western blotting was carried out using a standard protocol with appropriate antibodies (33). The primary antibodies included rabbit anti-nexilin (1:1,000; Abcam), mouse anti-MBP (1:2,000; Thermo Fisher Scientific, MA, USA), rabbit anti-thrombin receptor 1 (anti-PAR1; 1:1000; Millipore), mouse anti-RAP1 (1:1,000; Millipore), rabbit anti-pRAP1 (1:1,000; Millipore), rabbit anti-NG2 (1:1,000; Millipore), rabbit anti-Olig2 (1:1,000; Millipore), rabbit anti-β-actin (1:2,000; Zhongshanjinqiao, China), and rabbit anti-GAPDH (1:2,000; Goodhere, Hangzhou, China). The bands were probed with a chemiluminescence reagent kit (Advansta, Quebec, QC, Canada) and quantified by densitometry with ImageJ software (National Institutes of Health, Bethesda, MD, USA). Six times repeated Western blotting was performed for each protein. GAPDH or β-actin was used as the internal control for all experiments.

### Statistical Analysis

All quantitative values are given as the mean ± SD and were analyzed by SPSS13.0 or GraphPad Prism 5 software. Student’s *t*-test was used for the statistical analysis of *g*-ratios among two groups. The remaining data were statistically analyzed using one-way ANOVA followed by the Bonferroni multiple comparison method. *P* < 0.05 were considered statistically significant.

## Results

### OPC Proliferation and Migration to WM After SAH

The physiological parameters were similar among the groups (data not shown). No sham-operated mice died, while the total mortality in the SAH groups was 30.8% (15/84). In addition, 8 mice were excluded for having SAH grades that were too low (≤7) or too high (≥13), and the remaining 70 mice were included in the experiment.

**Figure 1:**
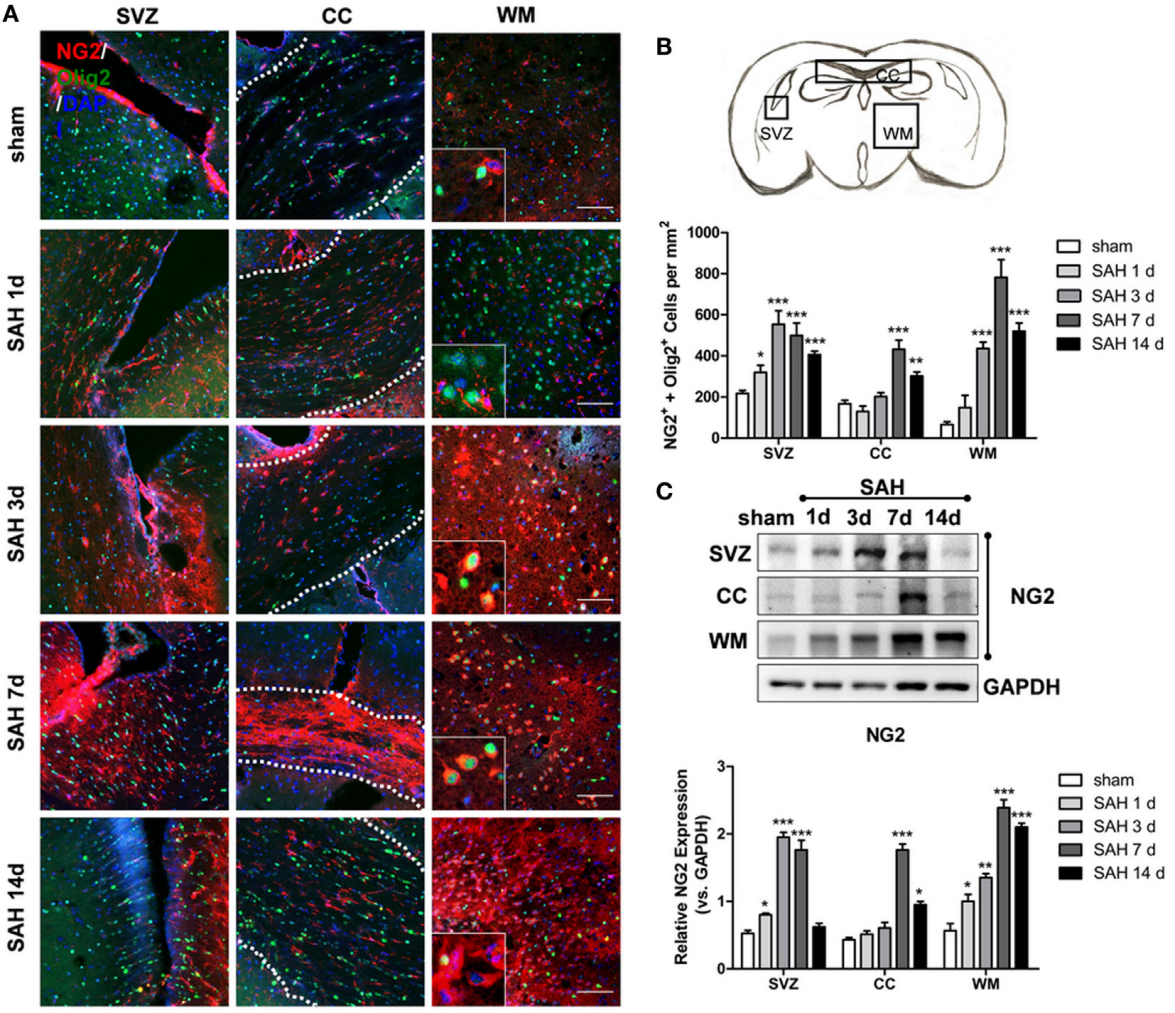
Oligodendrocyte progenitor cell proliferation and migration to WM after SAH. **(A)** Representative images of NG2- and Olig2-positive cells (20×) are shown. Scale bar = 50 µm. The anatomy drawing in the upper right corner indicates the relative areas. **(B)** Statistics of double-positive NG2 and Olig2 cells in the SVZ, CC, and WM of mouse brains at 1, 3, 7, and 14 days after SAH. **(C)** Western blot analysis of NG2 and GAPDH in distinct areas of the SVZ, CC, and WM at 1, 3, 7, and 14 days after SAH. The data are presented as the mean ± SEM, *n* = 6 for each group. *P < 0.05 versus the sham group, **P < 0.01 versus the sham group, ***P < 0.001 versus the sham group. SVZ, subventricular zone; CC, corpus callosum; WM, white matter; SAH, subarachnoid hemorrhage.

Double-positive NG2 and Olig2 (NG2^+^/Olig2^+^) cells indicate OPCs. Different regions of OPC proliferation were shown at 1, 3, 7, and 14 days after SAH (Figure 1A). Significant OPC proliferation peaked at 3 days post-SAH in the SVZ, followed by an increased number of NG2/Olig2-positive cells in the CC and WM that peaked at 7 days post-SAH. Moreover, the number of OPCs increased approximately 2.5-fold in the CC and 10-fold in WM at 7 days after SAH compared with that in the sham group (Figure 1B). NG2 expression was increased at 1, 3, and 7 days after SAH compared with that in the sham group, peaking at 3-day post-SAH. In contrast, NG2 expression was enhanced at 7 and 14 days after SAH in the CC and at 1, 3, 7, and 14 days after SAH in the SVZ; both peaks were observed at 7-day post-SAH (Figure 1C). Considering that OPCs in WM cannot proliferate *in situ* due to the lack of a stem cell pool (34), these data suggest that the elevated OPCs migrated from the SVZ to the CC and WM.

### Nexilin Expression in WM and OPCs after SAH

**Figure 2:**
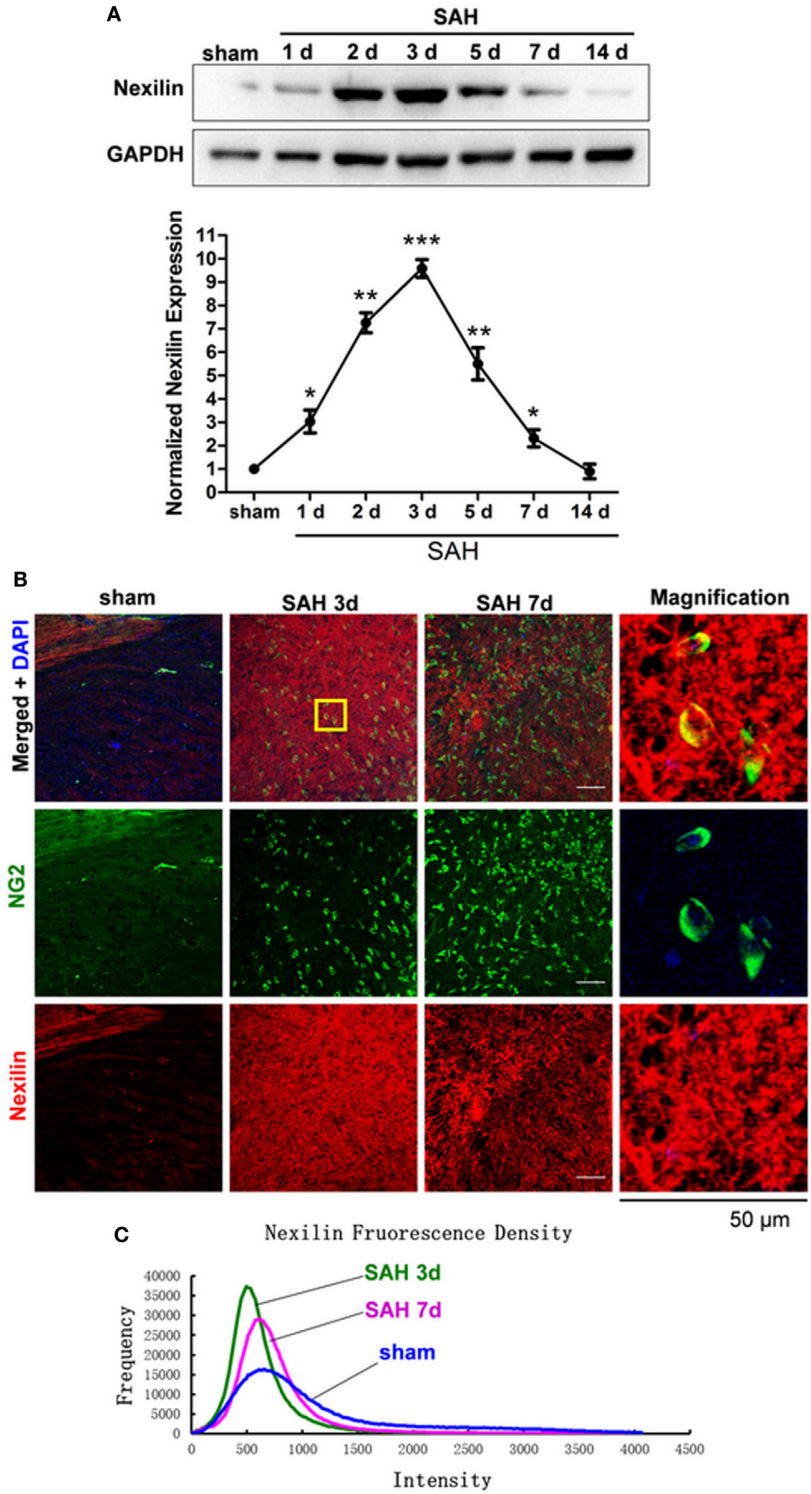
Nexilin expression in white matter and oligodendrocyte progenitor cells after SAH. **(A)** Western blot analysis of nexilin and GAPDH in white matter at 1, 2, 3, 5, 7, and 14 days after SAH. **(B)** Representative images of NG2- and nexilin-positive apoptotic neurons (20×) are shown. The magnified image on the right is enlarged five times. Scale bar = 50 µm. **(C)** Representative frequency curves of nexilin fluorescence intensities in the sham, SAH 3 days and SAH 7 days groups. The data are presented as the mean ± SEM, *n* = 6 for each group. *P < 0.05 versus the sham group, **P < 0.01 versus the sham group, ***P < 0.001 versus the sham group. SAH, subarachnoid hemorrhage.

Compared with that in the sham group, the protein expression of nexilin in WM was elevated on days 1, 2, 3, 5, and 7 after SAH, peaking at 3 days, showing a tendency to first rise and then fall (Figure 2A). Immunofluorescence analysis showed nexilin and NG2 cell co-expression in the WM after SAH (Figure 2B). Furthermore, the fluorescence density of nexilin was enhanced at 3 and 7 days after SAH compared with that in the sham group (Figure 2C).

### Genetic Inhibition of Nexilin Impedes OPC Migration and Remyelination After SAH

**Figure 3:**
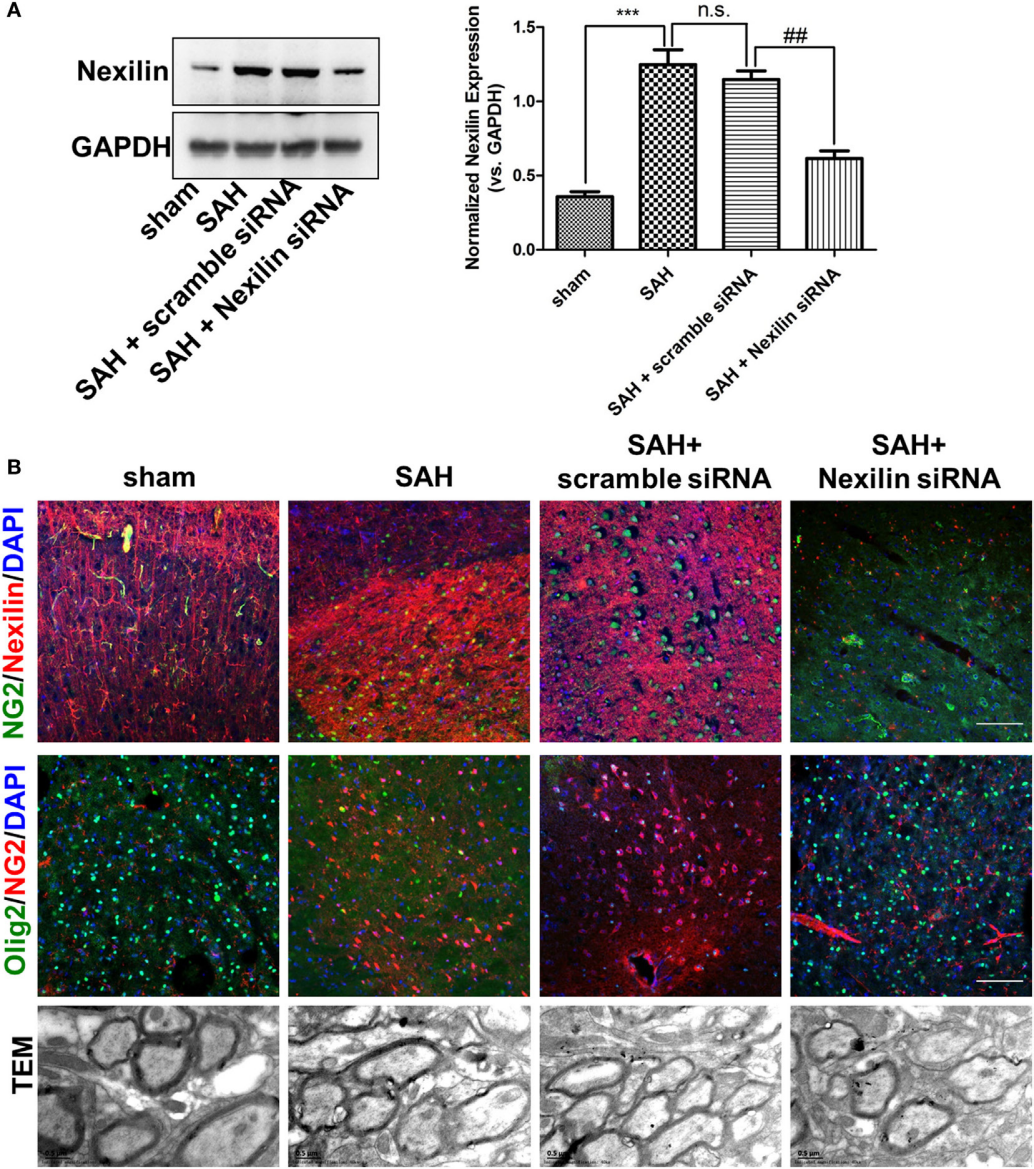

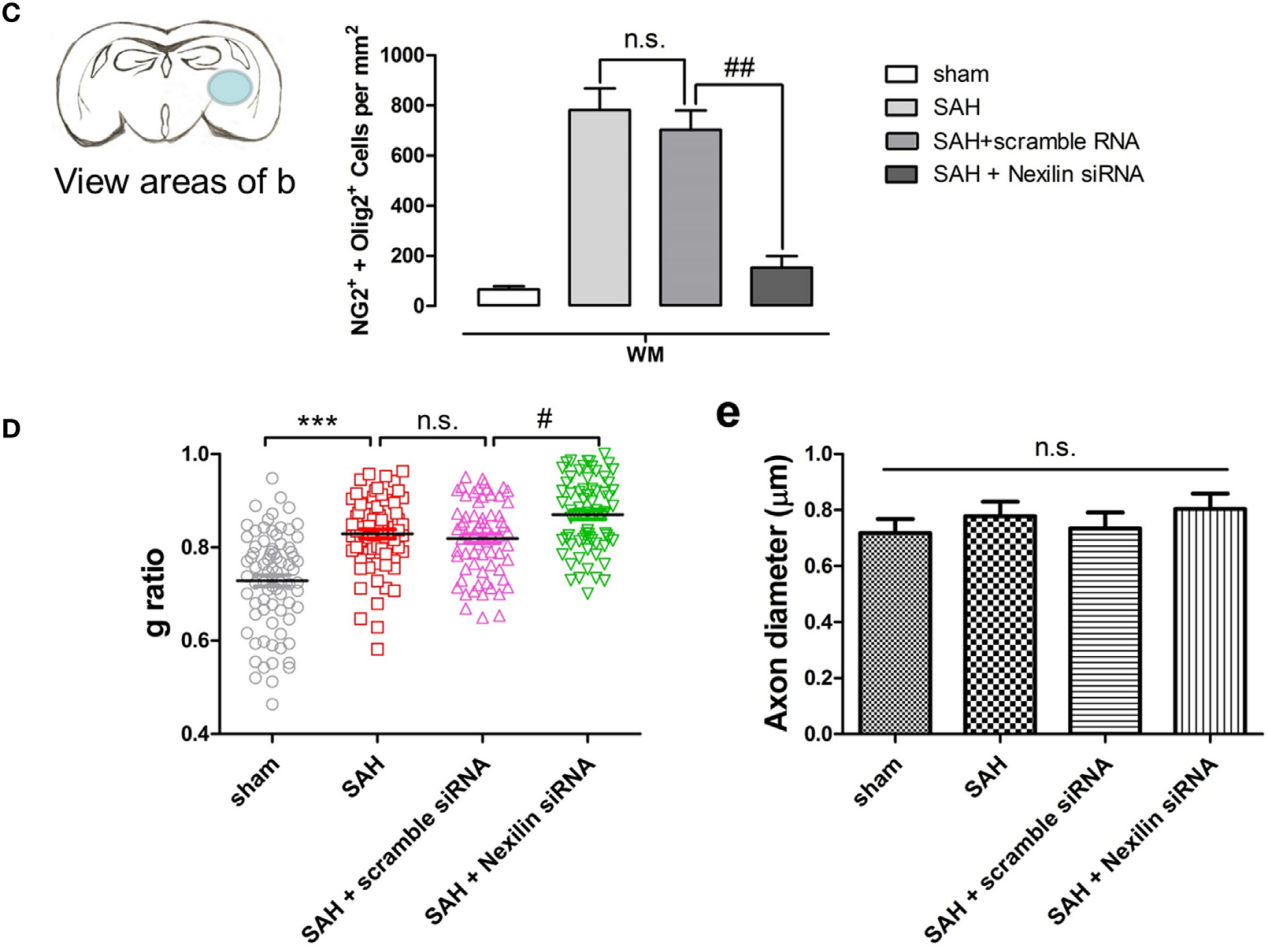
Genetic inhibition of nexilin impedes oligodendrocyte progenitor cell migration and remyelination after SAH. **(A)** Western blot analysis of nexilin and GAPDH in white matter (WM) at 3 days after SAH. The statistics histograms are shown on the right, *n* = 3 for each group. **(B)** Representative images of NG2/nexilin- and NG2/Olig2-positive cells and the myelin ultrastructure at 3 days after SAH are shown. **(C)** Statistics of double-positive NG2 and Olig2 cells in WM of mouse brains at 3 days after SAH, *n* = 3 for each group. **(D)** G-ratios representing the myelin thickness based on the TEM images, *n* ≥ 60 for each group. **(E)** Statistical histogram of the axon diameter (micrometer) of myelin based on the TEM images, *n* ≥ 60 for each group. The data are presented as the mean ± SEM; ***P < 0.001 versus the sham group, ^#^*P* < 0.05 versus SAH + scramble siRNA group, ^##^*P* < 0.01 versus SAH + scramble siRNA group, n.s. indicates no significance. SAH, subarachnoid hemorrhage.

To investigate the role of nexilin in OPC migration and remyelination after SAH, we administered a nexilin siRNA into the brains of SAH mice for 3 days. Western blotting and immunofluorescence analyses showed that the nexilin siRNA significantly reduced nexilin expression in WM at 3 days after SAH compared with that in the SAH + scramble group (Figure 3A). Genetically inhibiting nexilin using siRNA reduced the number of NG2^+^/Olig2^+^ cells in WM after SAH (Figures 3B,C). We examined the myelin ultrastructure, finding that SAH increased the *g*-ratio, suggesting a thinner myelin structure compared with that in the sham group (Figure 3B). Nexilin siRNA aggravated myelin thinning compared with that in the SAH + scramble siRNA group, whereas no significant differences in axon diameter were found in the sham and SAH groups (Figures 3D,E).

### Genetic Inhibition of Nexilin Impedes OPC Migration *In Vitro*

**Figure 4:**
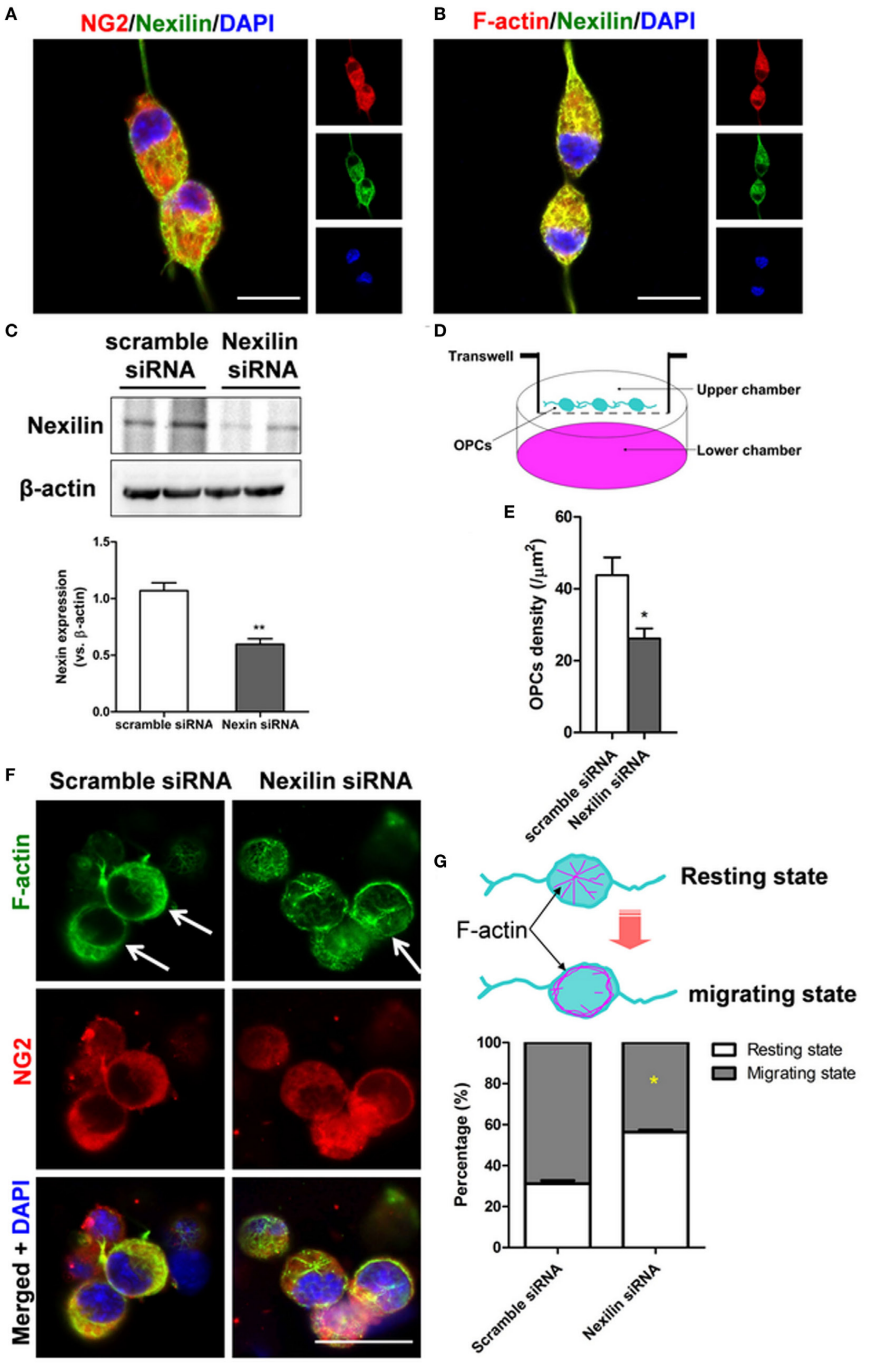
Genetic inhibition of nexilin impedes oligodendrocyte progenitor cell (OPC) migration *in vitro*. **(A,B)** Representative images of NG2/nexilin- and F-actin/nexilin-positive cells on confocal dishes are shown. Scale bar = 20 µm. **(C)** Western blot analysis of nexilin and β-actin in cultured OPCs. **(D)** Schematic diagram of transwell chamber assays. Platelet-derived growth factor (10 ng/ml) was added to the lower chamber to induce the migration of OPCs. **(E)** Statistical histogram of the number of OPCs per mm^2^, *n* = 6 for each group. **(F)** Representative images of NG2- and F-actin-positive cells treated with scramble siRNA or nexilin siRNA are shown. The white arrow indicates cells in the migratory state. **(G)** Upper panel, schematic diagram of OPCs in the resting and migrating states; lower panel, statistical histogram of the percentage of migratory state OPCs. *P < 0.05 versus scramble siRNA group, **P < 0.01 versus scramble siRNA group. SAH, subarachnoid hemorrhage.

To directly investigate the role of nexilin in OPC migration, nexilin siRNA was used to inhibit nexilin in cultured mouse OPCs. The cultured OPCs were NG2-positive and expressed nexilin (Figure 4A). Moreover, nexilin and F-actin were also co-expressed in OPCs (Figure 4B). Compared with that in the scramble siRNA group, nexilin expression in OPCs of the nexilin siRNA group was reduced by 50% (Figure 4C). We used a transwell chamber to detect the effects of nexilin on OPC migration. OPCs migrated to the lower chamber were counted, showing a significantly reduced OPC density (Figures 4D,E). Interestingly, inhibiting nexilin with siRNA caused F-actin rearrangement, as radially distributed F-actin (resting state) in the cytoplasm accumulated at the cell margins (migrating state) (Figure 4F). More precisely, nexilin inhibition reduced the percentage of OPCs in the migrating state (68.8 ± 2.5%) compared with that in the scramble siRNA group (43.6 ± 1.7%) (Figure 4G).

### PAR1/RAP1 Signaling Is Involved in OPC Migration After SAH

**Figure 5:**
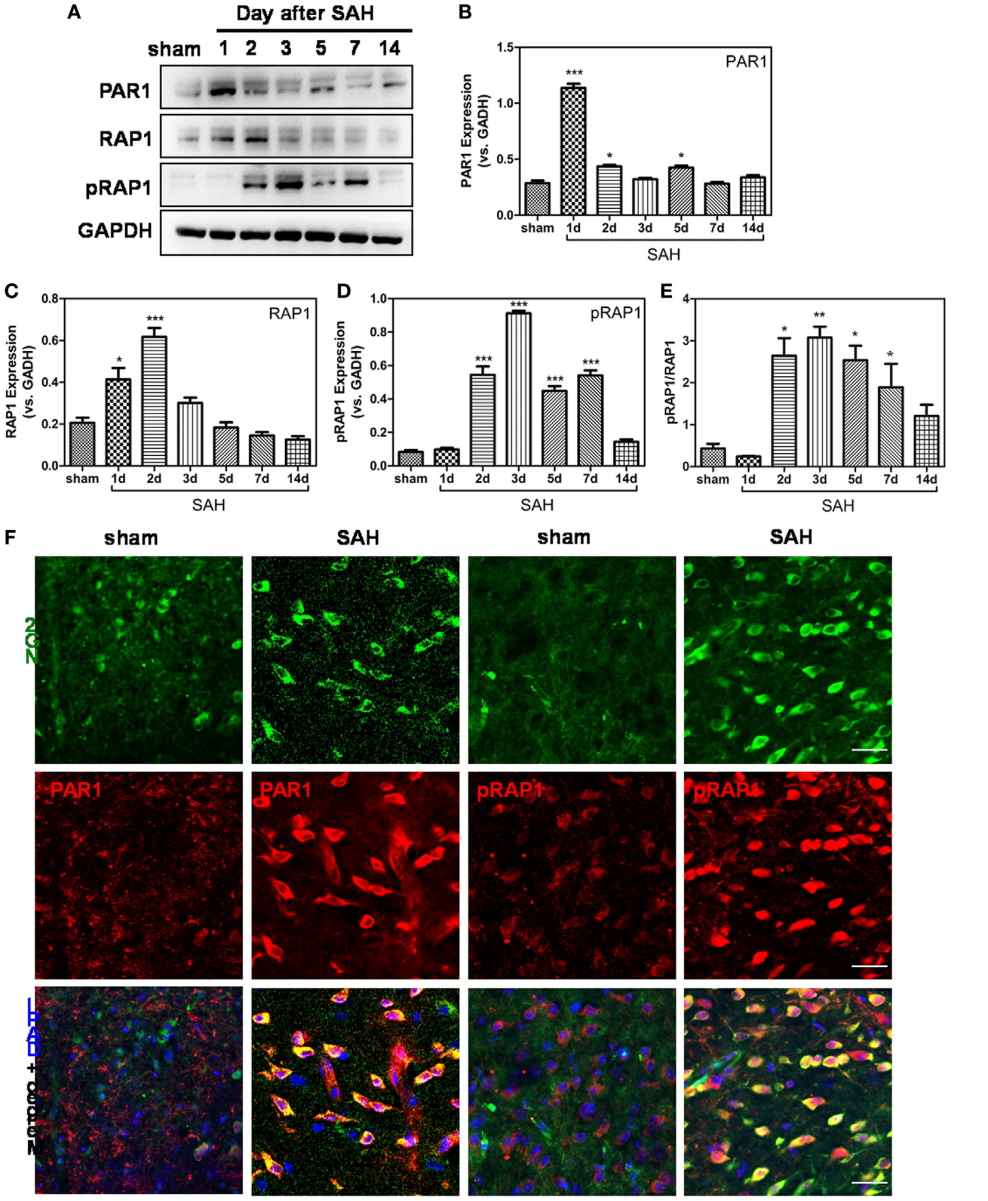
Protease-activated receptor 1 (PAR1)/Ras-proximate-1 (RAP1) signaling is involved in oligodendrocyte progenitor cell migration after subarachnoid hemorrhage (SAH). **(A)** Western blot analysis of PAR1, RAP1, phosphorylated RAP1 (pRAP1), and GAPDH in white matter at 1, 2, 3, 5, 7, and 14 days after SAH. **(B–E)** Respective statistical histograms of the normalized expression of PAR1, RAP1, pRAP1, and pRAP1/RAP1 ratio, *n* = 6 for each group. *P < 0.05 versus sham group, ***P < 0.001 versus the sham group. **(F)** Representative images of NG2/PAR1- and NG2/pRAP1-positive cells (20×) at 3 days after SAH are shown. Scale bar = 50 µm.

To explore the mechanisms underlying nexilin, we detected the expression of thrombin receptor signaling molecules at 1, 2, 3, 5, 7, and 14 days after SAH (Figure 5A). Compared with that in the sham group, PAR1 expression was enhanced at 1, 2, and 5 days after SAH and upregulated fivefold at 1-day post-SAH (Figure 5B). Meanwhile, RAP1 expression was shortly increased at 1 and 2 days after SAH, whereas no noticeable change was found at 3, 5, 7, and 14 days (Figure 5C). In contrast, the expressions of pRAP1 and pRAP1/RAP1 ratio were significantly upregulated at 2, 3, 5, and 7 days after SAH (Figures 5D,E). Furthermore, the expression of PAR1 and pRAP1 in NG2-positive cells was enhanced in WM at 2-day post-SAH compared with that in the sham group (Figure 5F).

### PAR1 Inhibition Enhances Nexilin Expression and Facilitates Remyelination After SAH

**Figure 6:**
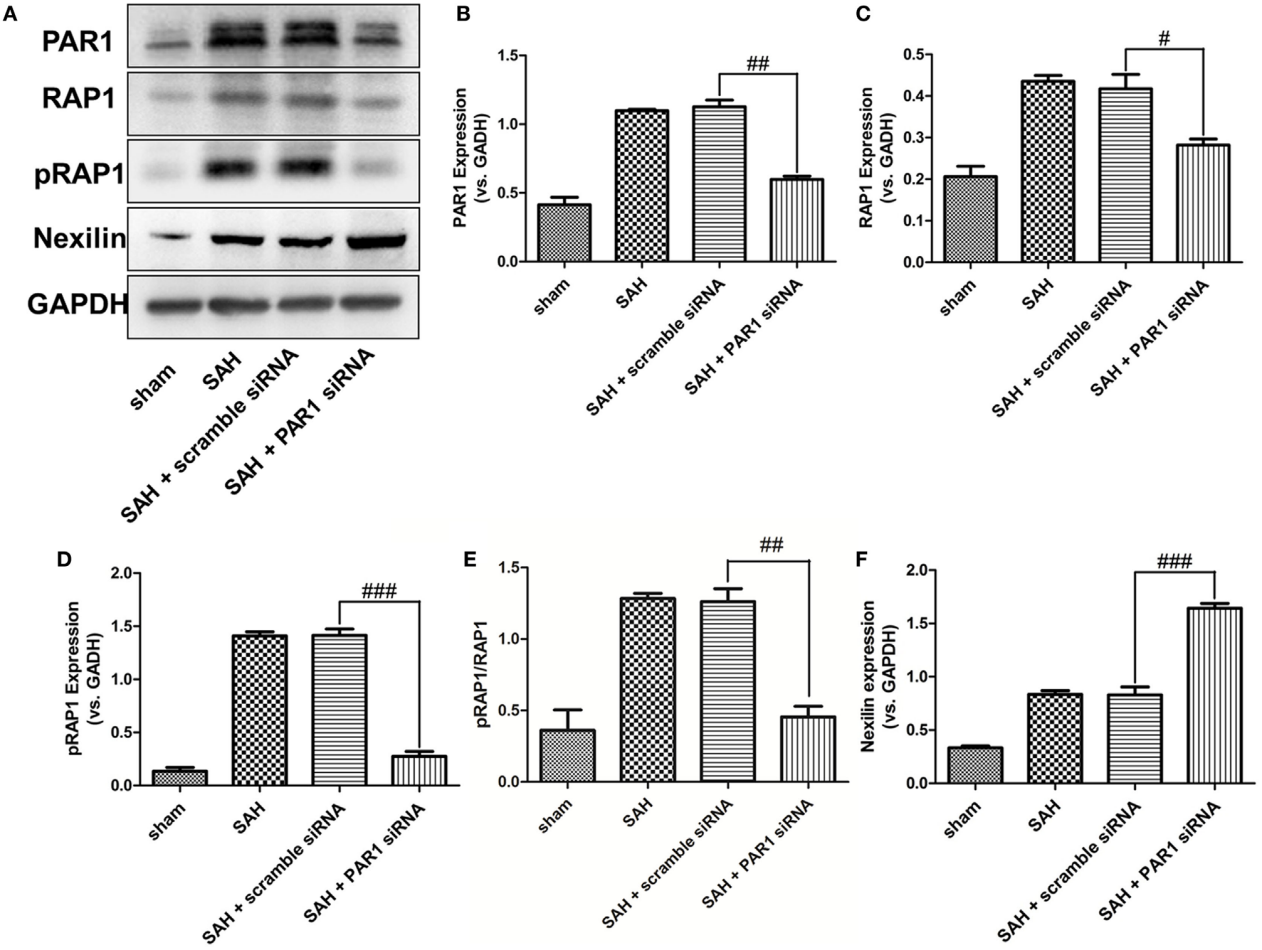

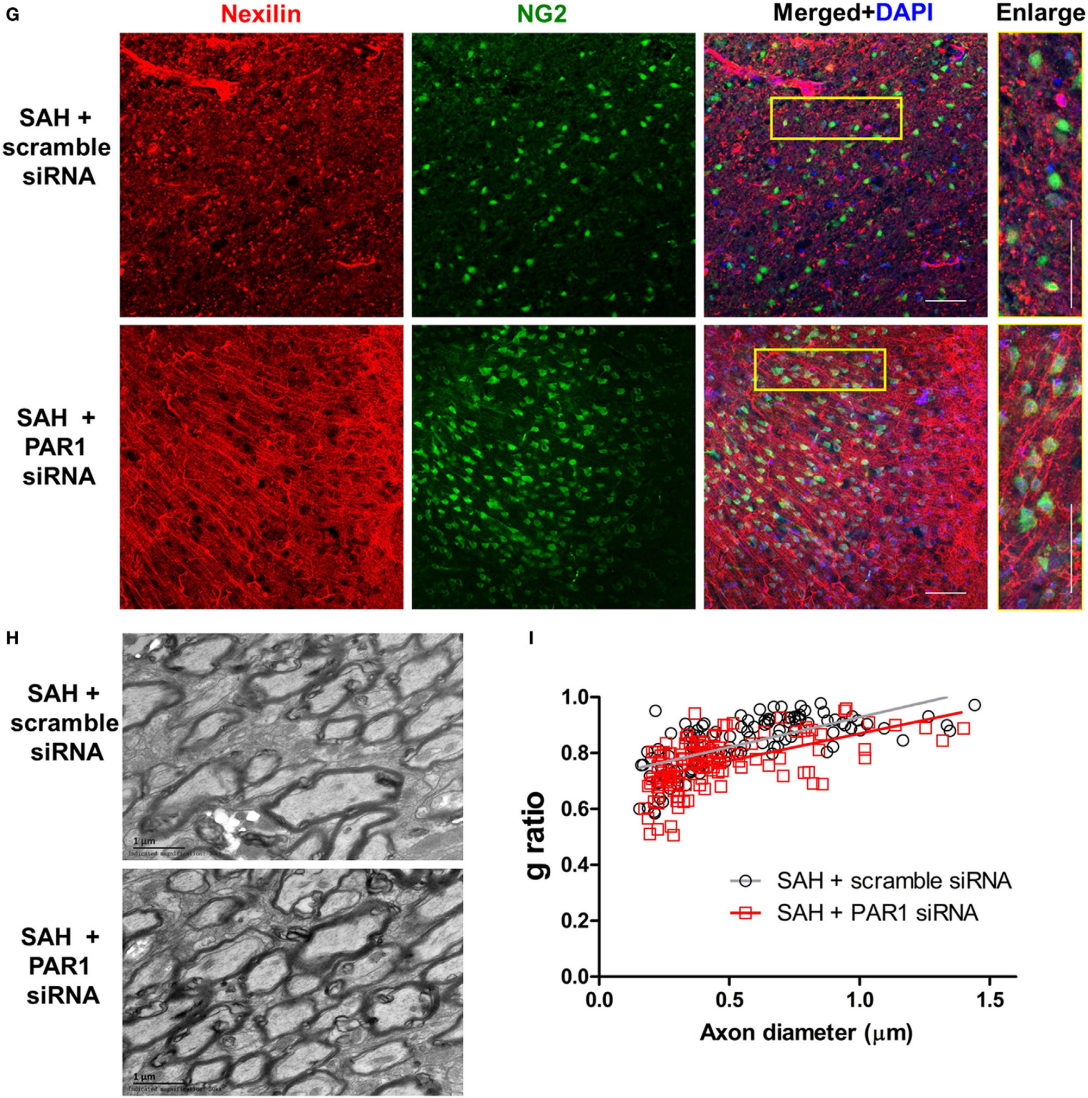
Inhibition of protease-activated receptor 1 (PAR1) enhanced nexilin expression and facilitated remyelination after subarachnoid hemorrhage (SAH). **(A)** Western blot analysis of PAR1, Ras-proximate-1 (RAP1), phosphorylated RAP1 (pRAP1), nexilin, and GAPDH in white matter at 3 days after SAH. **(B–F)** Respective statistical histograms of normalized expressions of PAR1, RAP1, pRAP1, nexilin, and pRAP1/RAP1 ratio, *n* = 6 for each group. ^#^*P* < 0.05 versus SAH + scramble siRNA group, ^##^*P* < 0.01 versus SAH + scramble siRNA group, ^###^*P* < 0.001 versus SAH + scramble siRNA group. **(G)** Representative images of NG2 and nexilin expression (20×) at 3 days after SAH are shown. Scale bar = 50 µm. **(H)** Representative transmission electron microscopy images of myelin in the SAH + scramble siRNA and SAH + PAR1 siRNA groups. **(I)** Respective *g*-ratios of the SAH + scramble siRNA (*y* = 0.2142*x* − 3.333, *r*^2^ = 0.4398) and SAH + PAR1 siRNA groups (*y* = 0.1943*x* − 3.473, *r*^2^ = 0.2849).

To clarify whether PAR1 signaling can regulate nexilin and SAH-induced demyelination, we used a PAR1 siRNA to intervene and detected relative protein expression levels at 2 days after SAH (Figure 6A). Inhibiting PAR1 by siRNA reduced the expressions of PAR1, RAP1, pRAP1, and pRAP1/RAP1 ratio compared with that in the scramble siRNA group (Figures 6B–E). The expression of nexilin, on the other hand, was further enhanced compared with that in the scramble siRNA group (Figure 6F). Additionally, inhibiting PAR1 increased nexilin expression in WM NG2-positive cells at 3 days after SAH (Figure 6G). Moreover, the administration of PAR1 siRNA (*R*^2^ = 0.2849) significantly reduced the *g*-ratio compared with that in the scramble group (*R*^2^ = 0.4398), suggesting that PAR1 inhibition restored remyelination after SAH (Figures 6H,I).

### TRAP Promotes Remyelination and Neural Functional Recovery After SAH

**Figure 7:**
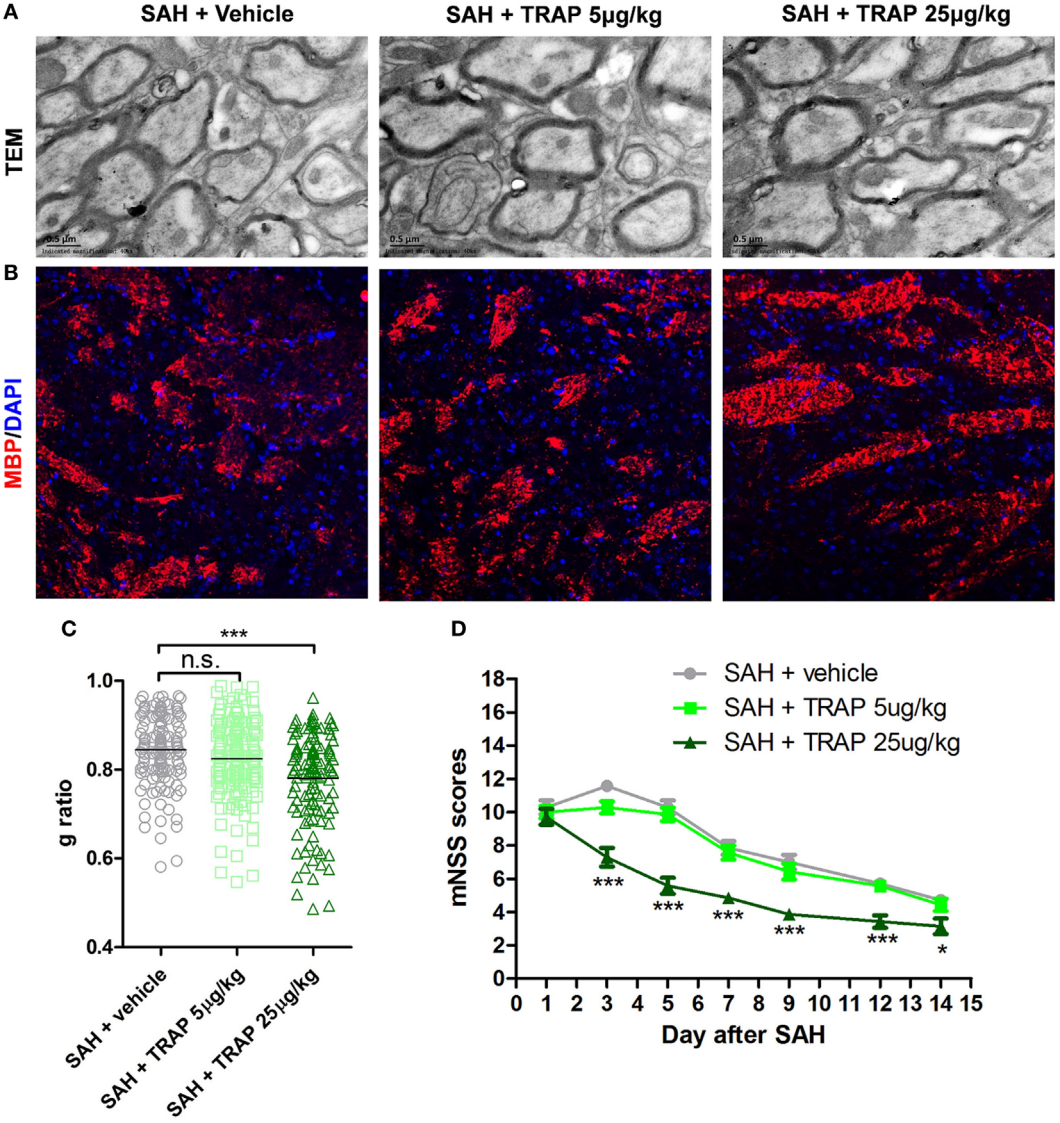
Thrombin receptor antagonist peptide promoted remyelination and neural functional recovery after subarachnoid hemorrhage (SAH). **(A,B)** Representative images of transmission electron microscopy and myelin basic protein (MBP) expression at 3 days after SAH are shown. Scale bar = 20 µm. **(C)** Respective myelin *g*-ratios, *n* = 125 for each group. **(D)** Respective modified neurological severity scale (mNSS) scores in each group at 1, 3, 5, 7, 9, 12, and 14 days after SAH, *n* = 10 for each group. The data are presented as the mean ± SEM. *P < 0.05 versus SAH + vehicle group, ***P < 0.001 versus SAH + vehicle group. n.s. indicates no significance.

To explore any available intervention strategies, we directly administered TRAP rather than small molecule inhibitors to inhibit PAR1 signaling after SAH because small molecule thrombin receptor inhibitors have thus far proven to be ineffective in clinical trials. Two concentrations of TRAP were used to analyze its effects on remyelination and MBP expression at 3 days after SAH (Figures 7A,B). The administration of 25 µg/kg TRAP reduced the *g*-ratio and enhanced MBP expression at 3 days after SAH, whereas no obvious difference was found between the SAH + vehicle and SAH + TRAP 5 µg/kg groups (Figure 7C). Furthermore, mNSS analysis, an evaluation indicator of motor and cognitive functions, showed that 25 µg/kg TRAP significantly alleviated SAH-induced neurological deficits at 1, 3, 5, 7, 9, 12, and 14 days after SAH, whereas no significant change was found in the 5 µg/kg TRAP group (Figure 7D).

## Discussion

**Figure 8:**
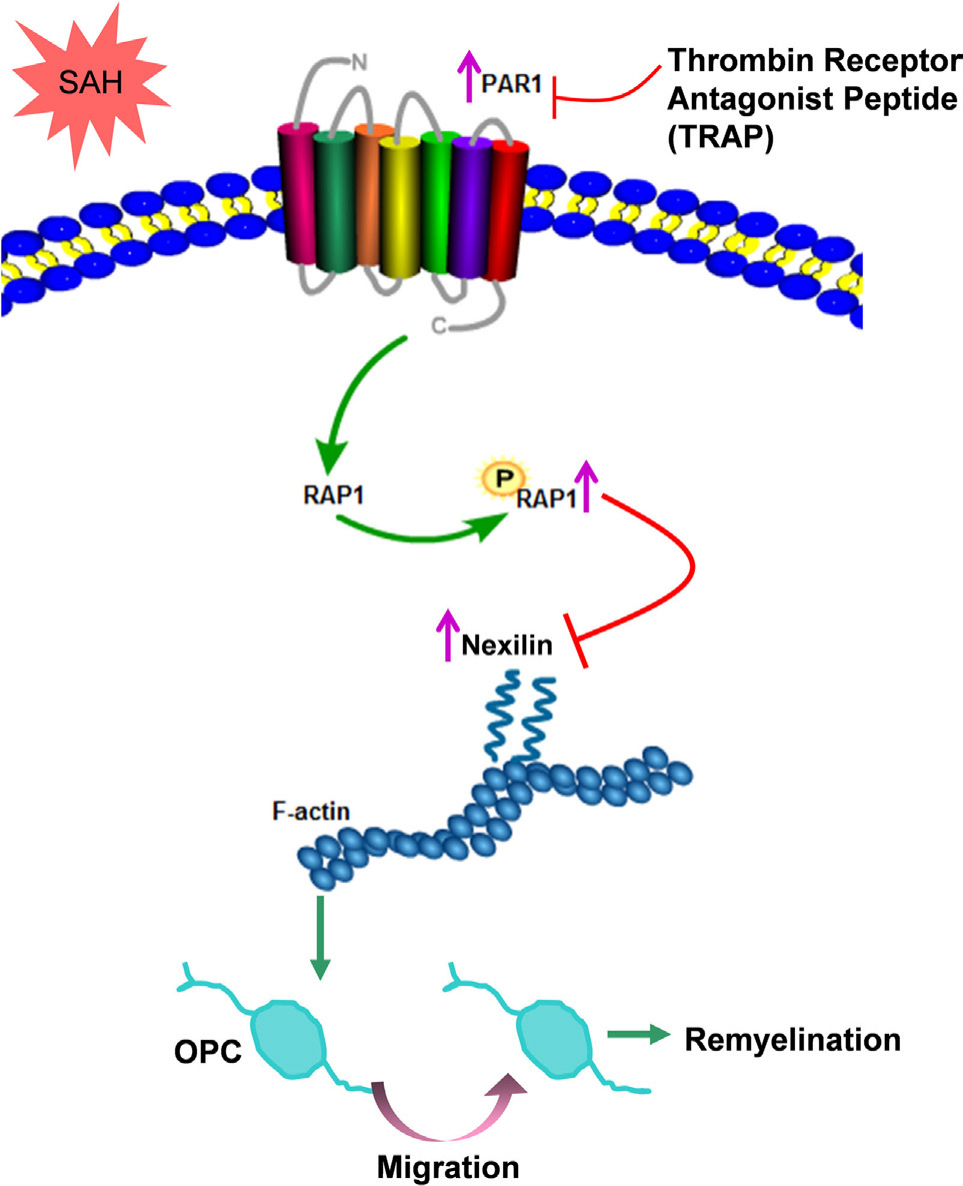
Schematic diagram of the present study. After subarachnoid hemorrhage (SAH), elevated nexilin levels facilitate F-actin rearrangement and then promote oligodendrocyte progenitor cell (OPC) migration and remyelination. Nexilin expression is negatively regulated by the activated thrombin receptor protease-activated receptor 1 (PAR1) on the OPC membrane, which is followed by the activation of downstream Ras-proximate-1 (RAP1) and the promotion of RAP1 phosphorylation. The suppression of PAR1 by thrombin receptor antagonist peptide (TRAP) inhibits PAR1 signaling but enhances nexilin and promotes OPC migration and remyelination after SAH.

Oligodendrocytes form myelin sheaths, which are essential for axons because they provide nutritional support and guarantee high-speed electrical impulse transmission (35). Myelin sheaths are also susceptible to multiple neurological disorders and leads to motor dysfunction, cognitive deficits, and other neurological impairments (36). OPCs are the main source for repairing damaged oligodendrocytes (37). Therefore, as the damage occurs, OPCs are recruited to the lesioned area, where they differentiate into mature remyelinating oligodendrocytes and engage in the formation of new myelin sheaths around axons (38). Herein, we found that OPC proliferation and migration occurred after SAH and determined that these processes were related to remyelination. Nexilin was demonstrated to be a key molecule regulating OPC migration by interacting with F-actin, which was negatively modulated by the PAR1 signaling pathway as shown in Figure 8. Given that no effective means to counter demyelinating injury after SAH have been elucidated (3), our work suggests that OPCs can serve as a therapeutic target for the acute stage of SAH-induced demyelination.

Some studies on ischemic stroke and intracerebral hemorrhage have shown the amounts of proliferating OPCs and the migration of OPCs to the lesion area across long distances (39, 40). However, whether SAH can induce OPC proliferation and migration has not been studied. Therefore, the present study appears to be the first to address the fate of oligodendrocyte-lineage cells after SAH in mice. Although the SVZ and CC are both the main sources of OPCs for remyelination (41), we found that significant OPC proliferation occurred in the SVZ earlier than that in the CC in the early stage after SAH. We speculated whether OPC proliferation in the CC started slower or whether these increased cells migrated from the SVZ. Furthermore, a previous study reported that newly generated OPCs in the SVZ cannot be recruited to the CC after chronic cerebral hypoperfusion (42). While the source of more OPCs in the CC is controversial and requires more study in the context of SAH, OPCs proliferating in WM are well recognized to migrate from the SVZ and CC rather than to proliferate *in situ*. In short, more OPCs in WM can considerably reflect their migration ability, as we have shown herein.

Oligodendrocyte progenitor cell migration is an extremely complex and precise event that is guided and directed by many different mechanisms (43). Positive signals that promote OPC migration mainly include PDGF signaling in development and NG2 signaling in adult remyelination (44). In contrast, negative signals that repel or stop OPC migration include netrin-1, chemokine CXCL1, SEMA3A, and Eph/ephrins, etc. (12). In addition, some researchers agree that contact with a preformed axonal tract is a main factor underlying OPC migration, and various adhesion molecules have been shown to mediate the migration of OPCs across astrocytic surfaces, extracellular matrices, and axonal tracts (45). Most of these emerging mechanisms that explore the molecular basis of migration have not been verified in SAH scenarios. In this study, thrombin receptor PAR1 signaling was found to be a negative cue that suppressed OPC migration and impeded remyelination following SAH. Indeed, this finding was supported by experiments performed on the spinal cord; however, the authors noted that PAR1 deletion promoted remyelination, probably by increasing OPC differentiation (23, 46). Whether PAR1 signaling also inhibits OPC differentiation after SAH should be studied in the future. The ability to promote and guide the migration of specific OPCs from the oligodendroglial cell pool could allow this cell population to be used for hemorrhagic WM repair, especially when considering the limiting factor of OPCs migrating to demyelinated lesions due to inhibited PAR1 signaling (47).

As previously stated, the thrombin-PAR1/RAP1 signaling pathway boost platelet aggregation and thus plays roles in hemostasis and thrombosis, which may raise the risk of rebleeding after SAH. Early rebleeding (within the first 6 h after the primary bleeding) is an important cause of death and disability following aneurysmal SAH (48). The mechanism underlying rebleeding remains elusive; however, decreases in thrombin–anti-thrombin and plasma factor XIII activities by less than 40%, associated with a latent clotting activity induced by thrombin generation, may increase the risk of rebleeding after an initial intracranial bleeding event (49). Whether the suppression of thrombin-PAR1/RAP1 signaling reduces clot stability in the acute phage of SAH needs further investigation in clinical and experimental studies. Substituting factor XIII in TARP-induced SAH protection may be one way to minimize the risk of rebleeding.

Elucidating the amounts of identified extracellular signals involved in OPC migration and intracellular cues that turn on cytoskeleton protein rearrangement is required to understand OPC migration (12). These factors are highly expressed in filopodial tips and could interact with the cytoskeleton or integrins, such as NG2 and hepatocytic growth factor, which have been proposed to act as motogenic cues for OPC migration (50, 51). However, their precise roles in OPC migration remain unclear, and more efforts are needed to characterize the details underlying this process. As a novel F-actin binding protein localized at the cell-matrix adherence junction, nexilin reportedly plays a pivotal role in cell migration. However, evidence of nexilin playing a similar role in OPC migration is lacking. Our study demonstrated that nexilin was expressed in OPCs and promoted OPC migration by interacting with the cytoskeleton F-actin protein. A previous study revealed the important effects of nexilin on cardiovascular diseases, including cardiomyopathy and endomyocardial fibroelastosis (52). Given that nexilin is highly expressed not only in OPCs but also in neurons and cerebral vasculatures after SAH (data not shown), the potential roles of nexilin in neural system disorders require more attention in the future.

However, some limitations of our study should be further addressed. First, we used a nexilin siRNA to inhibit nexilin both *in vivo* and *in vitro*. Considering the efficiency and toxicity of gene interference, a nexilin deletion (NEXN^−/−^) or even a conditional nexilin deletion (e.g., Pdgfra^cre/cre^Nexn^fl/fl^) in an OPC mouse model should be used to study the role of nexilin in OPC migration in the future. Second, we investigated the role of nexilin in OPC migration *in vitro* using classic transwell analysis. In our *in vivo* experimental design, we counted the numbers of OPCs in WM at different time points post-SAH instead of using a tracer to probe newly migrated OPCs in WM. Third, considering the limitation of intraventricular administrations, a single TRAP treatment was effective in the acute phage but may fail to alleviate WM injuries over a long period. Therefore, a pump buried in the lateral ventricle for continuous administration should be adopted to observe TRAP’s long-term efficacy after SAH.

In summary, our investigation provides new insights into the role of nexilin in OPC recruitment and remyelination following SAH. The protective effects of nexilin on OPC migration and SAH-induced demyelination are negatively modulated by the PAR1/RAP1 signing pathway. Our work provides a promising therapeutic target in WM repair and functional recovery after SAH.

## Ethics Statement

All experimental procedures were approved by the Ethics Committee of Southwest Hospital and performed in accordance with the guidelines in the National Institutes of Health Guide for the Care and Use of Laboratory Animals and reported by following the ARRIVE guidelines.

## Author Contributions

QL, HR, YC, and HF designed the experimental protocols. QL, HZ, PP, XR, SZ, JQ, and BL performed the experiments. YC, HR, QL, and HF carried out data analysis. QL and YC prepared and revised the manuscript. YC and HR gave the final approval of manuscript to be published.

## Conflict of Interest Statement

The authors declare that the research was conducted in the absence of any commercial or financial relationships that could be construed as a potential conflict of interest.

## Abbreviations

SAH, subarachnoid hemorrhage; WFNS, World Federation of Neurosurgical Societies; GCS, Glasgow Coma Scale; OPCs, oligodendrocyte progenitor cells; SEMA7F, semaphorin 7F; PDGF, platelet-derived growth factor; VEGF, vascular endothelial growth factor; SEMA3A, semaphorin 3A; CXCL1, chemokine (C-X-C motif) ligand 1; PAR1, protease-activated receptor 1; PLP, proteolipid protein; DAPI, 4’,6-diamidino-2-phenylindole, dihydrochloride.
